# Development and Validation of a Method for Determining the Quercetin-3-*O*-glucuronide and Ellagic Acid Content of Common Evening Primrose (*Oenothera biennis*) by HPLC-UVD

**DOI:** 10.3390/molecules26020267

**Published:** 2021-01-07

**Authors:** Tae Heon Kim, Hyun Young Shin, Soon Yeong Park, Hoon Kim, Dae Kyun Chung

**Affiliations:** 1 Graduate School of Biotechnology and Institute of Life Science and Resources, Kyung Hee University, Deogyeong-daero 1732, Giheung-gu, Yongin 17104, Korea; kimbob159@naver.com; 2 Department of Integrated Biomedical and Life Science, Korea University, Seoul 02841, Korea; shydud0629@korea.ac.kr; 3 Dain Natural Co., Ltd., 130-33, Gasong-ro, Pungse-myeon, Dongnam-gu, Cheonan 31216, Korea; rnd@dainnatural.com; 4 Skin-Biotechnology Center, Kyung Hee University, Gwanggyo-ro 147, Yeongtong-gu, Suwon 16229, Korea; 5 Graduate School of Biotechnology, Kyung Hee University, Deogyeong-daero 1732, Giheung-gu, Yongin 17104, Korea

**Keywords:** *Oenothera biennis*, standardization, quercetin-3-*O*-glucuronide, ellagic acid, method validation

## Abstract

Toward the standardization of common evening primrose (*Oenothera biennis)* sprout extract (OBS-E), we aimed to obtain indicator compounds and use a validated method. HPLC-UVD allowed simultaneous quantification of the indicator compounds quercetin-3-*O*-glucuronide and ellagic acid. The method was validated in terms of specificity, linearity, precision, accuracy, and limit of detection/limit of quantification (LOD/LOQ). High specificity and linearity was demonstrated, with correlation coefficients of 1.0000 for quercetin-3-*O*-glucuronide and 0.9998 for ellagic acid. The LOD/LOQ values were 0.486/1.472 μg/mL for quercetin-3-*O*-glucuronide and 1.003/3.039 μg/mL for ellagic acid. Intra-day and inter-day variability tests produced relative standard deviation for each compound of <2%, a generally accepted precision criterion. High recovery rate were also obtained, indicating accuracy validation. The OBS-E prepared using various concentrations of ethanol were then analyzed. The 50% ethanol extract had highest content of quercetin-3-*O*-glucuronide, whereas the 70% ethanol extract possessed the lowest. However, the ellagic acid content was highest in the 70% ethanol extract and lowest in the 90% ethanol extract. Thus, quercetin-3-*O*-glucuronide and ellagic acid can be used industrially as indicator compounds for *O. biennis* sprout products, and our validated method can be used to establish indicator compounds for other natural products.

## 1. Introduction

The evening primrose (genus *Oenothera*), belonging to the Onagraceae family, is a genus of approximately 145 species of herbaceous flowering plants distributed around the world. These plants are known to originate from tropical and temperate climate regions in South America, but have recently been observed in Asia, Europe, Africa, and Oceania where they have been used for edible and medicinal purposes [1]. In Korea, the leaves and sprouts of evening primrose are used to make Kimchi or some as a side dish [2], and reports have been published on their medicinal properties, such as their antioxidant [2,3], anti-inflammatory [4], antibacterial [5], anticancer [6,7], and anti-obesity [2] activities. Many types of *Oenothera* spp., such as *O. odorata*/*O. stricta*, *O. glazioviana*, *O. erythrosepala*, *O. biennis*, and *O laciniata* are now found in Korea [8]. *Oenothera biennis* (OB), the most commonly known evening primrose species worldwide, can be found in many countries and regions, including Korea, Japan, Australia, Britain, France, and Hawaii [9]. The most studied biological properties of OB are its antioxidant [3,10,11] and inflammatory [12,13] properties. However, most studies on OB have focused on the physiological activity or active ingredients, and investigation reported thus far into the standardization of OB, for example, to establish indicator compounds, are inadequate. Furthermore, although the leaves and sprouts of evening primrose are used for edible and medicinal purposes in Korea, most of the studies in the literature have been mainly focused on root and seed tissues.

**Figure 1:**
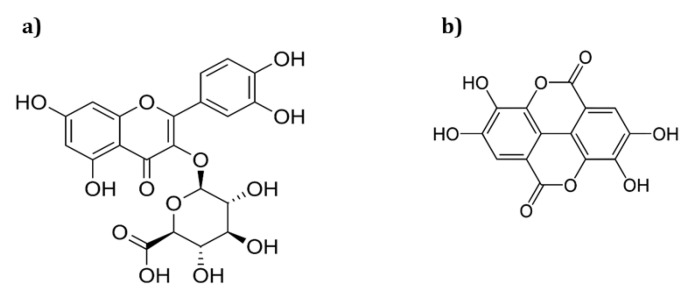
Chemical structure of (**a**) quercetin-3-*O*-glucuronide and (**b**) ellagic acid.

According to our preliminary results carried out by UHPLC-MS, *O. biennis* sprout ethanol extract (OBS-E) contained significant amounts of quercetin-3-*O*-glucuronide and ellagic acid (Supplementary Table S1), and the results are under review in other journal. Quercetin-3-*O*-glucuronide is a flavonol glycoside in which the acidic sugar glucuronoic acid is attached to the 3′-carbon of quercetin (Figure 1a). This compound is reported to have anti-inflammatory [14], anti-cancer [15], and antidepressant [16] efficacies. In an analytical study on quercetin-3-*O*-glucuronide, Nugroho et al. [16] compared its content in the leaves of various *Rubus* species using HPLC, and Ahmed et al. [17] evaluated its content in the aerial part of *Euphorbia schimperi* by a validated high-performance thin layer chromatography method. In addition, Kim et al. [18] demonstrated qualitative and quantitative determination of quercetin-3-*O*-glucuronide in various raspberry wines by UHPLC-DAD-QTOF/MS. Ellagic acid (Figure 1b), as a dimer of the simplest polyphenolic acid, gallic acid, is known to possess several physiological activities in humans, including antioxidant properties [19,20], anti-inflammatory properties [20,21], liver protection [22], and immunity enhancement [23]. Kim et al. [24] and Lee et al. [25], in analytical studies, determined the ellagic acid contents of natural substances—*Rubus coreanus* Miquel and *Nymphaea tetragona*, respectively—in Korea.

To apply and utilize a natural substance as a health functional ingredient, towards standardizing a natural substance, it is necessary to establish one or more indicator compounds, develop an analytical method, and validate the developed method. Analytical method validation comprises a series of processes that confirm that the selected analytical method is reproducible and provides reliable results that are fir for the intended purpose [26]. In addition, such validation procedures have received general recognition as means of determining the validity of an analysis method for quality control of functional foods and pharmaceutical products [27]. Therefore, the aim of this study was to develop an analytical method for the simultaneous determination of the quercetin-3-*O*-glucuronide and ellagic acid contents of OBS-E by HPLC-UV method, and to validate the method in terms of specificity, linearity, precision, accuracy, and limit of detection (LOD)/limit of quantification (LOQ). In addition, the contents of quercetin-3-*O*-glucuronide and ellagic acid of various OBS extracts prepared using different ethanol concentrations were compared, as a proof-of-concept, using basic data, for the standardization of OBS as a health functional ingredient.

## 2. Results and Discussion

This study was aimed at the development of a validated method for simultaneously determining indicator compounds, such as quercetin-3-*O*-glucuronide and ellagic acid, in OBS-E. The simultaneous determination of quercetin-3-*O*-glucuronide and ellagic acid was validated in terms of specificity, linearity, LOD/LOQ, precision, and accuracy.

### 2.1. Specificity and Linearity

**Figure 2:**
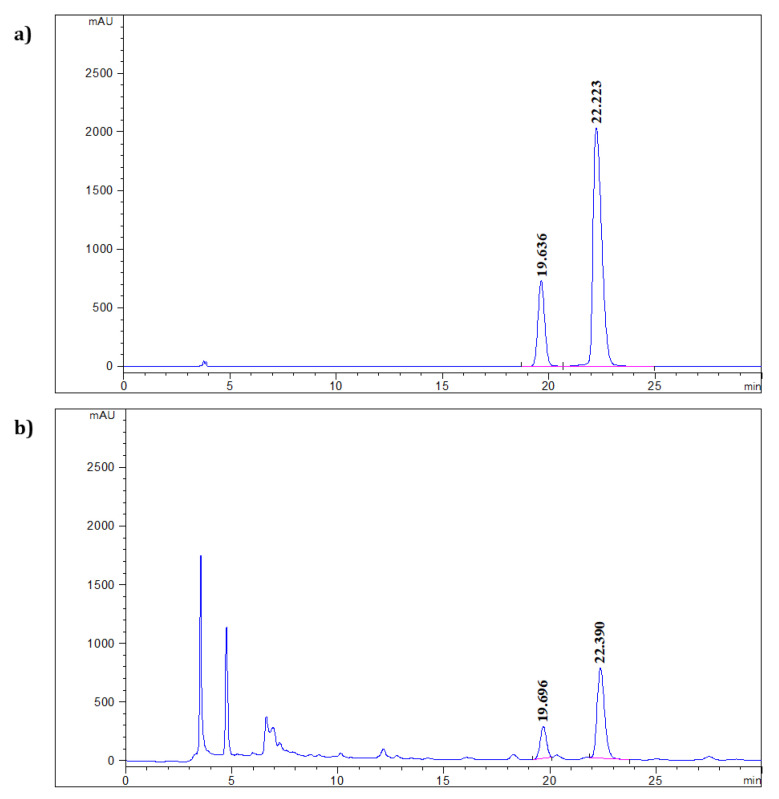
HPLC chromatogram of (**a**) mixed standard of quercetin-3-*O*-glucuronide (19 min) and ellagic acid (22 min), and (**b**) ethanol extract of *O. biennis* sprout.

First, two peaks were clearly identified in the chromatogram of the mixed standard solution, which were identified as the quercetin-3-*O*-glucuronide (19.636 min) and ellagic acid (22.223 min) (Figure 2a). As shown in Figure 2b, two main peaks were also confirmed at the same retention times (19.696 min and 22.390 min, respectively) in the mixed standard chromatogram.

**Figure 3:**
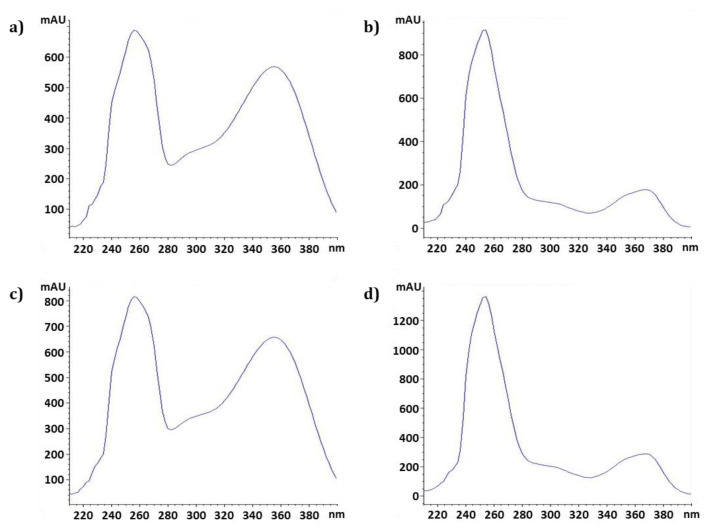
UV spectrum structure of quercetin-3-*O*-glucuronide and ellagic acid. (**a**) Quercetin-3-*O*-glucuronide reference; (**b**) Ellagic acid reference; (**c**,**d**) *O. biennis* sprout ethanol extract.

Furthermore, the UV spectra corresponding to both peaks in the sample chromatogram were consistent with those in the mixed standard chromatogram, validating the specificity of the method for the detection of the two compounds (Figure 3). In other words, these results indicated that our simultaneous method is a specific and completely distinguishable method for the analysis of quercetin-3-*O*-glucuronide and ellagic acid in OBS-E.

Next, calibration curves were obtained by the external standard method, using six concentrations of the standard mixtures, with three injections per concentration (Supplementary Figure S2). Chromatogram peak areas were plotted against the known concentrations of the standard solutions to establish the calibration equations, and linear regression equations were calculated via the least squares method (Supplementary Figure S3). This simultaneous method showed linear regressions at concentrations from 14.625 to 468 μg/mL for quercetin-3-*O*-glucuronide and from 15 to 480 μg/mL for ellagic acid, the correlation coefficient (R^2^) of the regression equation presented were 1.0000 and 0.9998, respectively (Table 1). These results indicate that for both compounds, the simultaneous analysis method developed has excellent linearity in the indicated concentrations ranges.

### 2.2. Limit of Determination and Limit of Quantification

Although the residual standard deviation can be calculated in various ways [26], we used the standard deviation of the y-intercept in the regression equation as this value. Consequently, the LOD/LOQ values were found to be 0.5 and 1.5 μg/mL, respectively, for quercetin-3-*O*-glucuronide and 1.0 and 3.0 μg/mL, respectively, for ellagic acid (Table 1). In a recent study on the development of a validated method for quercetin-3-*O*-glucuronide quantification in *Cuphea glutinosa*, an R^2^ value of 0.9999 was reported, and LOD/LOQ values were 8.41/25.51 μg/mL [28]. Thus, the LOD/LOQ values for this method were approximately 17 fold higher than those of our simultaneous determination method, suggesting that our method can be used more effectively to detect and quantify small amounts of quercetin-3-*O*-glucuronide. In addition, Assunção et al. [29] reported a validated method for which the LOD/LOQ values for ellagic acid were 0.66/2.22 μg/mL values that are similar to those we measured for the present method.

### 2.3. Precision and Accuracy

In order to validate our simultaneous method against criteria for precision, which is defined as the degree of agreement (scatter) among measured values for the same sample, the mixed standard was analyzed at three different concentrations to verify the repeatability. Three arbitrary concentrations of two standard compounds were selected and the residual standard deviation (RSD, %) were calculated from the results of repeated measurements for each concentration. In addition, the measurement was carried out three times on the same day to obtain the intra-day variability and three times on different days to obtain the inter-day variability, to compare the difference between the measured contents of the solutions with known concentrations relative to the variability of the analysis procedure.

The results showed that RSDs for quercetin-3-*O*-glucuronide at concentrations of 93.6, 187.2, and 374.4 μg/mL were 0.56, 0.11, and 0.16%, respectively, for the intra-day measurements, and 0.68, 0.26, and 0.51%, respectively, for the inter-day measurements (Table 2). These results indicate that the precision criterion for the determination of quercetin-3-*O*-glucuronide concentration is acceptable because the RSD is below 2%, which corresponds to the criterion recommended by the International Council for Harmonisation of Technical Requirements for Pharmaceuticals for Human Use (ICH) guidelines [30]. Meanwhile, the RSDs of ellagic acid at 96, 192, and 384 μg/mL were 0.46, 0.28, and 0.36%, respectively, for the intra-day measurement, and 2.02, 1.37, and 1.33%, respectively, for inter-day measurements (Table 2). The finding that the RSD values corresponding to the inter-day variability were slightly but significantly higher than in corresponding to the intra-day variability for ellagic acid suggests that this compound could be less stable over time after being dissolved. Nevertheless, our method for ellagic acid quantification, with an average RSD of 1.57% was verified according to the ICH precision criterion.

To validate the accuracy of the method, that is, to assess how close the values measured using the method are to the true values, known concentration of the two standard compounds were added to the OBS-E, and the percentage recovery was calculated by repeated measurement of the analytes. For quercetin-3-*O*-glucuronide, the percentage recovery for the three concentrations 93.6, 187.2, and 374.4 μg/mL confirmed the high accuracy of the method, with a mean recovery rate of 102.6% being measured (Table 3). To verify the accuracy for ellagic acid, the three independent concentrations 96, 192, and 384 μg/mL were used, and the mean recovery was confirmed to be 105.8% (Table 3). Consequently, our results confirm that the developed method for quercetin-3-*O*-glucuronide and ellagic acid quantification is validated in terms of both precision and accuracy.

### 2.4. Quantification of Quercetin-3-O-glucuronide and Ellagic Acid in Various O. biennis Sprout Extracts

Finally, our validated method was applied to the determination and comparison of quercetin-3-*O*-glucuronide and ellagic acid contents as indicator compounds in various OBS extracts. Five OBS extracts were prepared at different ethanol concentrations (0, 30, 50, 70, and 90 *v*/*v*%), and the obtained contents of quercetin-3-*O*-glucuronide and ellagic acid are presented in Table 4. The various OBS-E were found to contain quercetin-3-*O*-glucuronide and ellagic acid contents of 16.5–27.7 mg/g and 1.9–15.4 mg/g, respectively. Among the five extracts, the 50% ethanol extract showed the highest content of quercetin-3-*O*-glucuronide (27.7 mg/g), whereas for the 70% ethanol extract, showed the lowest content (16.5 mg/g) was obtained. In contrast, the ellagic acid content was the highest in the 70% ethanol extract, whereas the lowest content was found in the 90% ethanol extract. Interestingly, for the 70% ethanol extract, quite different levels of quercetin-3-*O*-glucuronide and ellagic acid were measured, compared to other extracts, suggesting that further experiments, such as an investigation of the compound–activity relationship are needed in the near future.

Granica et al. [31] investigated and identified 39 polyphenols from 50% ethanol extracts of the aerial parts of OB with UHPLC fingerprinting and tandem mass spectrometry analyses. In addition, Fecker et al. [32] recently found, by HPLC-UV analysis, that a 70% ethanol extract of the aerial parts of OB contained gallic acid, caffeic acid, epicatechin, coumaric acid, ferulic acid, rutin and rosmarinic acid. Wang et al. [33] isolated the ethyl acetate fraction from a 50% methanol extract of OB seed and identified gallic acid, procyanidin B3, catechin, and methyl gallate, along with aldose reductase inhibitory activity, using HPLC fingerprinting and proton NMR microscopy. De la Paz et al. [34] reported the composition and content of fatty acids, sterols, triterpene alcohols, squalenes, and phenols in OB extracted by low temperature pressurization. However, most of these studies were concerned with the aerial parts of OB, or its seed oil, whereas our study was the first investigation that successfully detected and quantified secondary metabolites extracted from the sprouts of OB. Therefore, it can be concluded that quercetin-3-*O*-glucuronide and ellagic acid can be used industrially as indicator compounds for products prepared using OBS, and our validated method can be used in the drug and health functional food industries. Furthermore, the approach can be used more widely to establish indicator compounds for other natural substances.

## 3. Materials and Methods

### 3.1. Reagents

Quercetin-3-*O*-glucuronide (CAS No. 22688-79-5) and ellagic acid (CAS No. 476-66-4) were obtained from the Natural Product Institute of Science and Technology (Anseong, Korea), accurately weighed and dissolved in dimethylsulfoxide (DMSO; Junsei Chemical Co., Ltd., Tokyo, Japan) to form 100 mg/mL stock solutions. All chemicals used in HPLC in the mobile phases were of HPLC grades purchased from Honeywell International Inc. (Charlotte, NC, USA).

### 3.2. Preparation of Standard Solutions

A mixed standard solution was prepared by adding the same volume of each stock solution, before adequate dilution with pure water. Consequently, the final concentration of DMSO was 1% in each mixed standard solution. The diluted standard mixture was filtered through polyvinyl difluoride (PVDF) syringe filters (0.2 μm; Pall Life Sciences, Ann Arbor, MI, USA) before being used for analysis.

### 3.3. HPLC-UVD Instrumentation and Analysis Method

An HPLC system (1200 series; Agilent Technologies, Inc., Palo Alto, CA, USA) coupled with a YMC-Triart C18 column (250 × 4.6 mm, 5 μm; YMC Co., Ltd., Kyoto, Japan) and a DAD unit (D1315D; Agilent Technologies, Inc.) at a wavelength of 257 nm was used in this study. The column temperature was set to 35 °C, and the injection volume was 20 μL. The flow rate was 0.8 mL/min. The gradient program for the mobile phase combined with water containing 2% formic acid (A) and acetonitrile containing 2% formic acid (B) as follows: 0→2 min (30→35% B), 2→8 min (35→42% B), 8→25 min (42→45% B), 25→30 min (45→30% B). The initial mobile phase condition was equilibrated for 10 min to ensure the reproducibility of the analysis.

### 3.4. Validation of Simultaneous Quercetin-3-O-glucuronide and Ellagic Acid Content Analysis Method

To validate the simultaneous analysis, we referred to the relevant ICH guideline [28]. The validation parameters were specificity, linearity, precision, accuracy, and LOD/LOQ.

#### 3.4.1. Specificity

The retention times and corresponding UV spectra for OBS-E and the standard reference were compared to validate whether the peaks indicated the presence of the same compounds.

#### 3.4.2. Linearity

The standard solutions were diluted the following concentrations. Quercetin-3-*O*-glucuronide: 14.625, 29.25, 58.5, 117, 234, and 468 μg/mL. Ellagic acid: 15, 30, 60, 120, 240, and 480 μg/mL. The diluted solutions were introduced to the HPLC system in triplicate, and a linear regression line was drawn for each compound using the concentrations and peak areas of the reference. The correlation coefficient (R^2^) of the regression equation was obtained to validate linearity parameter.

#### 3.4.3. Precision

To validate the precision parameter of the simultaneous analysis method, intra-day and inter-day variability were evaluated. The mixed standard solution of quercetin-3-*O*-glucuronide and ellagic acid was used to prepare three different dilutions of the standard solutions at concentrations in the range of 93.6–374.4 μg/mL and 96–384 μg/mL, respectively. To validate the intra-day and inter-day variability, the standard solutions were analyzed in triplicate, on the same day and on three different days, respectively.

#### 3.4.4. Accuracy

In order to assess accuracy, the mixed standard solution of quercetin-3-*O*-glucuronide and ellagic acid was used to prepare three different dilutions of the standard solutions, at concentrations in the range of 93.6–374.4 μg/mL and 96–384 μg/mL, respectively. To validate accuracy of the method, the diluted standard solutions were added to OBS-E and the resultant mixture was analyzed. The percentage recovery values for quercetin-3-*O*-glucuronide and ellagic acid were obtained from these results.

#### 3.4.5. Limit of Detection and Limit of Quantification

From the slope (*S*) and residual standard deviation (*σ*) of the regression equation from the diluted mixed standard solutions of quercetin-3-*O*-glucuronide and ellagic acid, LOD and LOQ values were obtained using the following Equations (1) and (2).

LOD = 3.3*σ/S* (1)

LOQ = 10*σ/S* (2)

### 3.5. Preparation of O. biennis Extracts Using Various Concentrations of Ethanol

Fresh evening primrose sprouts harvested in Gyeonggi province (Korea) in 2019 were used in this study. Internal transcribed spacer (ITS) and the US National Center for Biotechnology Information (NCBI) database analyses showed that the DNA sequence of our evening primrose sprouts had a 100% correspondence with that of OB (MT610948.1) (Supplementary Figure S1 and Table S2). Dried OBS obtained from Dain Natural Co, Ltd. (Seoul, Korea) was ground using a pulverizing machine (Hankookmc Co., Ltd., Incheon, Korea) and then extracted by refluxing with seven volumes of ethanol of various concentrations for 24 h at room temperature. The extract was filtered through a filtering cloth (10 μm, FilterTech Co., Ltd., Daejeon, Korea) to remove non-soluble particles, and then evaporated using a rotary vacuum evaporator (Eyela, Tokyo, Japan). After drying in a heating oven (Daesan Machinery, Hwaseong, Korea) at 70 °C for 48 h, the dried extracts were powdered using a pulverizing machine to obtain the OBS-E prepared using various concentrations of ethanol.

## 4. Conclusions

In the present study, quercetin-3-*O*-glucuronide and ellagic acid were selected as indicator compounds and a novel HPLC-UV method was developed and validated for precise and accurate determination of their contents in OBS extracts. When assessed against validation criteria, including specificity, linearity, precision, accuracy, and LOD/LOQ, our developed simultaneous method was shown to be acceptable. Using the validated method, the quercetin-3-*O*-glucuronide and ellagic acid contents of OBS extracts prepared using various concentrations of ethanol were determined. The OBS extracts were found to contain 16.5–27.7 mg/g and 1.9–15.4 mg/g of quercetin-3-*O*-glucuronide and ellagic acid, respectively. Consequently, we concluded that quercetin-3-*O*-glucuronide and ellagic acid can be used as indicator compounds for OBS-E, and our validated method can be used industrially for the standardization of products prepared using OBS-E.

## Figures and Tables

**Table 1:** Results of linearity regression, correlation coefficient, LOD, and LOQ for quercetin-3-*O*-glucuronide and ellagic acid.

Standard	Regression Equation	R^2^	Residual STD (*σ*)	Calibration Curve Slope (*S*)	LOD (μg/mL)	LOQ (μg/mL)
Quercetin-3-*O*-glucuronide	33.9200χ + 16.3234	1.0000	5.0	34.0	0.5	1.5
	33.9749χ + 16.3269	1.0000				
	33.9666χ + 7.6711	1.0000				
Integration (*n* = 3)	33.9538χ + 13.4405	1.0000				
Ellagic acid	118.7395χ + 322.2313	0.9997	36.3	119.4	1.0	3.0
	120.2432χ + 249.6622	0.9999				
	119.2289χ + 285.3886	0.9998				
Integration (*n* = 3)	119.4039χ + 285.7607	0.9998				

The mean ± standard deviation of peak retention time was 19.6 ± 0.1 min for quercetin and 22.3 ± 0.1 min for ellagic acid, respectively. *σ*, residual standard deviation; *S* = calibration curve slope; Limit of detection (LOD) = 3.3 × *σ*/*S*; Limit of quantification (LOQ) = 10 × *σ*/*S*.

**Table 2:** Intra-day and inter-day variabilities of quercetin-3-*O*-glucuronide and ellagic acid.

Standard	Conc. (μg/mL)	Intra-Day Variability (*n* = 3)		Inter-Day Variability (*n* = 3)	
		Mean ± SD	RSD (%)	Mean ± SD	RSD (%)
Quercetin-3-*O*-glucuronide	93.6	97.3 ± 0.5	0.6	97.7 ± 0.7	0.7
	187.2	194.2 ± 0.2	0.1	193.8 ± 0.5	0.3
	374.4	386.3 ± 0.6	0.2	387.2 ± 2.0	0.5
Ellagic acid	96.0	104.5 ± 0.5	0.5	104.2 ± 2.1	2.0
	192.0	210.3 ± 0.6	0.3	209.3 ± 2.9	1.4
	384.0	416.1 ± 1.5	0.4	415.2 ± 5.5	1.3

Conc., concentration; RSD, relative standard deviation.

**Table 3:** Results of accuracy validation for quercetin-3-*O*-glucuronide and ellagic acid.

Standard	Conc. (μg/mL)	Observed (μg/mL)	Recovery (%)
		Mean ± SD	Mean ± SD
Quercetin-3-*O*-glucuronide	93.6	96.0 ± 1.0	102.5 ± 1.0
	187.2	193.3 ± 0.7	103.3 ± 0.4
	374.4	381.3 ± 1.4	101.8 ± 0.4
Average (*n* = 9)			102.6 ± 0.8
Confidence interval (95%)			101.9–103.2
Ellagic acid	96.0	101.0 ± 0.6	105.2 ± 0.6
	192.0	204.2 ± 3.6	106.3 ± 1.9
	384.0	406.7 ± 4.3	105.9 ± 1.1
Average (*n* = 9)			105.8 ± 1.2
Confidence interval (95%)			104.9–106.8

Conc., concentration.

**Table 4:** Quantification of quercetin-3-*O*-glucuronide and ellagic acid in the evening primrose extract depending on extraction solvent.

Sample	Quercetin-3-*O*-glucuronide (mg/g)		Ellagic Acid (mg/g)	
	Mean ± SD	RSD (%)	Mean ± SD	RSD (%)
0% EtOH	24.6 ± 0.0	0.1	2.4 ± 0.1	5.5
30% EtOH	25.3 ± 0.0	0.1	2.8 ± 0.1	3.7
50% EtOH	27.7 ± 0.5	1.9	3.3 ± 0.1	2.0
70% EtOH	16.5 ± 0.0	0.2	15.4 ± 0.1	0.3
90% EtOH	24.9 ± 0.1	0.4	1.9 ± 0.2	10.6

RSD, relative standard deviation.

## Supplementary Materials

The following are available online, Figure S1: (a) Test article used in this study and (b) PCR amplification of test article by ITS DNA analysis. M: 1Kb(+), 1: test article, N: negative control; Figure S2: Overlaid chromatogram of standard mixture comprising quercetin-3-*O*-glucuronide and ellagic acid at various concentrations; Figure S3: Calibration curve of (a) quercetin-3-*O*-glucuronide and (b) ellagic acid. Table S1: Putative identification of four major secondary metabolites in OBS-E using UHPLC-MS; Table S2: Genetic identification of test article by ITS DNA analysis.

## Sample Availability

Samples of the compounds *O. biennis* sprout extract are available from the authors.

## Abbreviations

Dimethylsulfoxide	DMSO
High performance liquid chromatography-diode array detector	HPLC-DAD
High performance liquid chromatography-ultraviolet detector	HPLC-UVD
International Council for Harmonisation of Technical Requirements for Pharmaceuticals for Human Use (ICH)	ICH
Internal transcribed spacer	ITS
*Oenothera biennis*	OB
*Oenothera biennis* sprout	OBS
*Oenothera biennis* sprout extract	OBS-E
Residual standard deviation	RSD
Ultra-high performance liquid chromatography-diode array detector-quadrupole time-of-flight mass spectrometry	UHPLC-DAD-QTOF/MS
Ultra-high performance liquid chromatography-mass spectrometry	UHPLC-MS
