# Supplementary material for: Development and Validation of a Method for Determining the Quercetin-3-O-glucuronide and Ellagic Acid Content of Common Evening Primrose (Oenothera biennis) by HPLC-UVD
Source: Molecules. 2021 Jan 7;26(2):267. doi: 10.3390/molecules26020267 (PMC7827709; doi:10.3390/molecules26020267)
Supplement: Supplementary file 1 [file molecules-26-00267-s001.pdf]

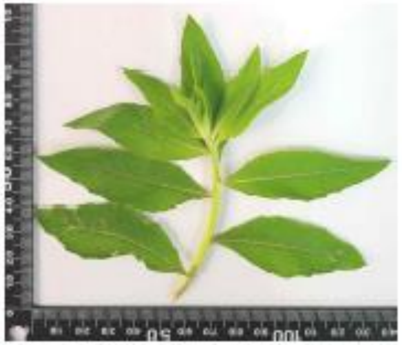

a)

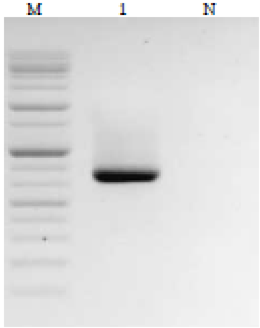

b)

Supplementary Figure S1. a) Test article and b) PCR amplification of test article by ITS DNA analysis. M: 1Kb(+), 1: test article, N:negative control.

Supplementary Table S1. Putative identification of four major secondary metabolites in OBS-E using UHPLC-MS.

RT (min)	*m/z*	Ionized form	Calculated formula	MS/MS (*m/z*)	Δppm	Identification
1.95	169.0000	($[M-H]^-$)	C7H5O5	124	-1.594	Gallic acid
6.68	301.0833	($[M-H]^-$)	C14H5O8	184,200,229,256,284	-1.990	Ellagic acid
6.94	479.0788	($[M+H]^+$)	C21H19O13	303	-1.694	Quercetin 3-glucuronide
7.78	463.0841	($[M+H]^+$)	C21H19O12	287	-1.656	Luteolin 7-glucuronide

Supplementary Table S2. Genetic identification of test article by ITS DNA analysis

Sample	Gene bank No. (NCBI)	Identification	Similarity
Evenig primrose	MT610948.1	*Oenothera biennis*	704/704 (100%)

Supplementary results

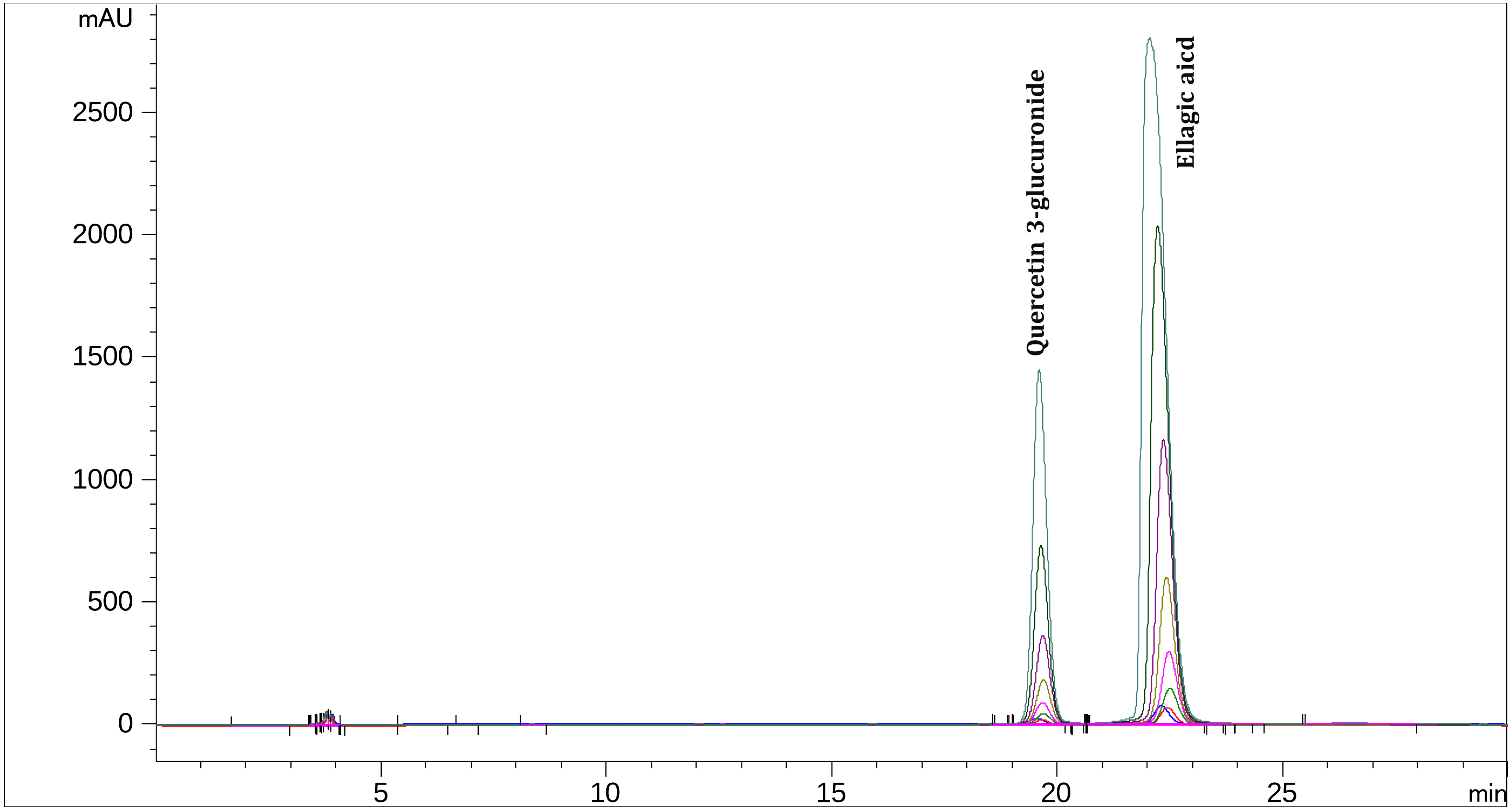

Supplementary Figure S2. Overlaid chromatogram of standard mixture comprising quercetin-3-O-glucuronide and ellagic acid at various concentrations. Quercetin-3-O-glucuronide: 14.625, 29.25, 58.5, 117, 234, and 468 µg/mL; Ellagic acid: 15, 30, 60, 120, 240, and 480 µg/mL.

Supplementary results

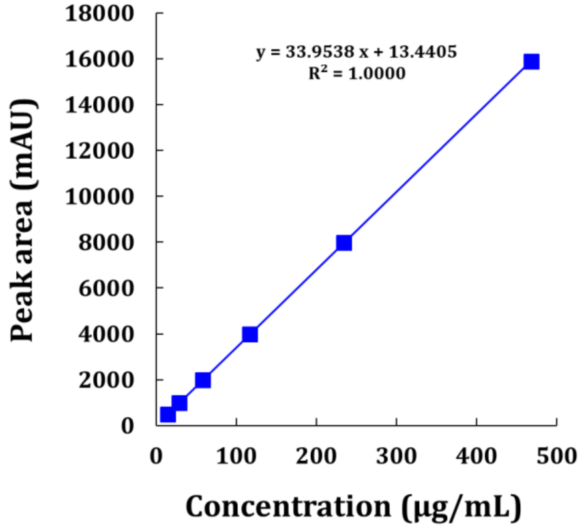

a)

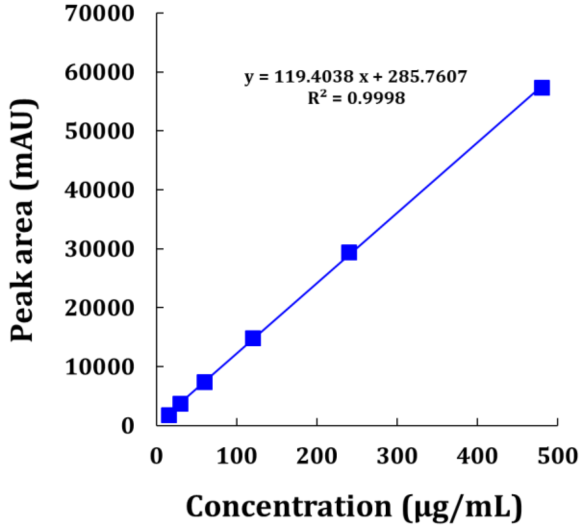

b)

Supplementary Figure S3. Calibration curve of a) quercetin-3-O-glucuronide and b) ellagic acid. Quercetin-3-O-glucuronide: 14.625, 29.25, 58.5, 117, 234, and 468 µg/mL; Ellagic acid: 15, 30, 60, 120, 240, and 480 µg/mL.

Supplementary results
